# Adjustment of creatinine clearance for carboplatin dosing in Calvert's formula and clinical efficacy for lung cancer

**DOI:** 10.1002/cam4.6235

**Published:** 2023-06-23

**Authors:** Takahiro Hatta, Tetsunari Hase, Toru Hara, Tomoki Kimura, Eiji Kojima, Takashi Abe, Yoshitsugu Horio, Yasuhiro Goto, Naoya Ozawa, Naoyuki Yogo, Hirofumi Shibata, Tomoya Shimokata, Tetsuya Oguri, Masashi Yamamoto, Kiyoshi Yanagisawa, Masahiko Ando, Yuichi Ando, Masashi Kondo, Makoto Ishii, Yoshinori Hasegawa

**Affiliations:** ^1^ Department of Respiratory Medicine Nagoya University Graduate School of Medicine Nagoya Japan; ^2^ Department of Respiratory Medicine Anjo Kosei Hospital Anjo Japan; ^3^ Department of Respiratory Medicine and Allergy Tosei General Hospital Seto Japan; ^4^ Department of Respiratory Medicine Komaki City Hospital Komaki Japan; ^5^ Department of Respiratory Medicine Ogaki Municipal Hospital Ogaki Japan; ^6^ Department of Thoracic Oncology Aichi Cancer Center Hospital Nagoya Japan; ^7^ Department of Respiratory Medicine Fujita Health University School of Medicine Toyoake Japan; ^8^ Department of Clinical Oncology and Chemotherapy Nagoya University Hospital Nagoya Japan; ^9^ Department of Education and Research Center for Community Medicine Nagoya City University Graduate School of Medical Sciences Nagoya Japan; ^10^ Department of Respiratory Medicine Nagoya Ekisaikai Hospital Nagoya Japan; ^11^ Division of Molecular and Cancer Medicine, Faculty of Pharmacy Meijo University Nagoya Japan; ^12^ Center for Advanced Medicine and Clinical Research Nagoya University Hospital Nagoya Japan; ^13^ National Hospital Organization, Nagoya Medical Center Nagoya Japan

**Keywords:** carboplatin, Cockcroft–Gault formula, creatinine clearance, glomerular filtration rate, non‐small‐cell lung cancer

## Abstract

**Background:**

The Cockcroft–Gault formula is commonly used as a substitute for glomerular filtration rate (GFR) in Calvert's formula for carboplatin dosing, where adjusting serum creatinine measured using the enzymatic method with 0.2 mg/dL has been suggested in Japan. However, the effects of these adjustments on efficacy in patients with non‐small‐cell lung cancer remain unknown.

**Methods:**

We conducted a post hoc analysis of the PREDICT1 study (CJLSG1201), a multicenter prospective observational trial of carboplatin–pemetrexed. Glomerular filtration rate values in Calvert's formula were back‐calculated from the administered dosages of carboplatin and the reported value of the target area under the curve. We estimated the serum creatinine adjustments and divided the patients into crude and adjusted groups.

**Results:**

Patients in the crude group (*N* = 169) demonstrated similar efficacy to those in the adjusted group (*N* = 104) in progression‐free survival (PFS) and overall survival (OS) (hazard ratio [HR], 1.02; 95% confidence interval [CI], 0.76–1.35; *p* = 0.916 vs. HR, 0.87; 95% CI, 0.65–1.17; *p* = 0.363), with higher grade 3–4 hematologic toxicity. Among patients aged ≥75 years, the crude group (*N* = 47) showed superior efficacy compared with the adjusted group (*N* = 17) in PFS and OS (HR, 0.37; 95% CI, 0.20–0.69; *p* = 0.002 vs. HR, 0.43; 95% CI, 0.23–0.82; *p* = 0.010).

**Conclusions:**

Serum creatinine adjustment may be associated with similar efficacy compared to the crude serum creatinine value. In older patients, the adjustment should be cautiously applied owing to the potential for reduced efficacy.

## Graphical abstract

Adjustment of serum creatinine measured using the enzymatic method with 0.2 mg/dL in creatinine clearance calculation using the Cockcroft–Gault formula is associated with similar clinical efficacy and a low incidence of toxicity compared with those of non‐adjusted values in patients with preserved renal function eligible for carboplatin–pemetrexed treatment. However, adjustments should be used with special caution in older patients owing to the potential for reduced efficacy.

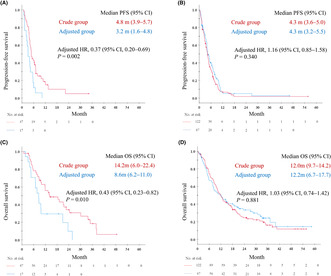

## 1 INTRODUCTION

Carboplatin, a platinum anticancer agent, is commonly used for many human cancer types, including lung and gynecological cancers.^1^ As carboplatin clearance strongly correlates with glomerular filtration rate (GFR), and the antitumor effect and toxicity of carboplatin correlate with the area under the blood concentration‐time curve (AUC), carboplatin dosage is determined using Calvert's formula based on the target AUC and GFR.^2^, ^3^, ^4^ The GFR in this formula was initially measured using the ^51^Cr‐EDTA method^3^; however, it was complicated and not used in clinical practice. The Cockcroft–Gault formula has been developed for estimating creatinine clearance (CCr) and is commonly used as a substitute for GFR in Calvert's formula.^5^, ^6^ Other equations for estimating the GFR have also been proposed and evaluated.^7^, ^8^, ^9^, ^10^, ^11^

The serum creatinine (SCr) in the Cockcroft–Gault formula was initially measured using the Jaffé method, which was approximately 0.1–0.3 mg/dL higher than the value measured using the enzymatic or isotope dilution mass spectrometry method.^12^ Therefore, in the Cockcroft–Gault formula, estimated CCr (eCCr) based on SCr value measured using these methods could theoretically overestimate GFR, resulting in a potential overestimation of carboplatin doses.^13^, ^14^, ^15^ Thus, in Japan, adding 0.2 mg/dL to the measured SCr value has been suggested following the implementation of the enzymatic method to achieve a pharmacokinetically accurate target carboplatin AUC.^16^, ^17^, ^18^ Several studies comparing the measured GFR value with the estimated value have been conducted for cancer patients^16^, ^17^, ^19^, ^20^, ^21^, ^22^, ^23^, ^24^, ^25^; however, the effects of these adjustments on clinical benefit in patients with non‐small‐cell lung cancer (NSCLC) treated with carboplatin have not been sufficiently investigated. In addition, carboplatin is commonly used in older patients as it is less emetogenic and safer for patients with impaired renal function than cisplatin; in Japan, carboplatin–pemetrexed treatment is administered as standard therapy to patients aged ≥75 years with non‐squamous NSCLC.^26^ However, the Cockcroft–Gault formula using the SCr value measured using the Jaffé method underestimates GFRs in older patients^27^, ^28^; therefore, carboplatin dosing with adjustments in these patients may affect their clinical outcomes.

Here, we conducted a post hoc analysis of a multicenter prospective observational trial of carboplatin–pemetrexed treatment in patients with non‐squamous NSCLC to investigate the effect of SCr adjustment on chemotherapeutic efficacy and toxicity.

## 2 METHODS

### 2.1 Study design and patients

This was a post hoc analysis study of the PREDICT1 trial (CJLSG1201), a multicenter prospective observational trial of carboplatin–pemetrexed combination therapy followed by maintenance pemetrexed as first‐line treatment (University Medical Information Network in Japan number: UMIN000008476).^29^ The inclusion criteria were age ≥ 20 years, clinical Stage III disease not receptive to definitive radiotherapy, Stage IV or recurrent disease, no prior chemotherapy, presence of measurable lesions according to Response Evaluation Criteria in Solid Tumors (RECIST) guideline version 1.1,^30^ and adequate organ function. Patients with previous chest radiotherapy or other primary cancer were excluded. The primary study protocol^29^ was approved by the institutional ethics committee of each participating institution, and all patients provided written informed consent. This post hoc analysis was approved by the Ethics Review Committee of Nagoya University Graduate School of Medicine (No. 2018‐0386).

### 2.2 Treatment assessments

Computed tomography tumor assessment was repeated every 6 weeks for 36 weeks and, after that, every 9 weeks. All responses were assessed according to RECIST criteria. A confirmatory evaluation was required after at least 4 weeks if a complete or partial response was observed. The response was considered a stable disease if it was confirmed and sustained for ≥6 weeks after initiation of chemotherapy. Progression‐free survival (PFS) was defined as the time from the initiation of study treatment to the date of confirmation of progressive disease or date of death from any cause, whichever occurred first. Overall survival (OS) was defined as the time from initiation of chemotherapy until death from any cause. Toxicities were graded according to National Cancer Institute Common Terminology Criteria for Adverse Events version 4.0.

### 2.3 Assessment of GFR by back‐calculation based on actual carboplatin dose and reported AUC value

In the PREDICT1 study,^29^ 500 mg/m^2^ pemetrexed was administered. Both target AUC (5–6) and the method for estimating GFR in Calvert's formula were at the investigator's discretion. In addition, dose reduction was also conducted at the investigator's discretion. The GFR values were back‐calculated based on the actual administered carboplatin dosage and reported value of the target AUC using modifying Calvert's formula, as follows:^3^

$$\begin{array}{c} \text{Back‐calculated}\;\mathrm{GFR}\;\left(\text{bGFR}\right)\;\left(\mathrm{mL}/\min\right)\\ \;=\text{actual carboplatin dose}\;\left(\mathrm{mg}\right)/\text{target}\;\mathrm{AUC}–25. \end{array}$$

### 2.4 Estimation of SCr adjustment

SCr levels were measured using an enzymatic method at all institutions. We hypothesized that carboplatin dosage was determined based on Calvert's formula, where GFR was substituted by CCr calculated using the Cockcroft–Gault formula, as follows:^5^

$$\begin{array}{c} \text{eCCr}\;\left(\mathrm{mL}/\min\right)=\left\{\left(140-\mathrm{age}\;\left(\text{years}\right)\right)\times \text{weight}\;\left(\mathrm{kg}\right)\right\}\\ \;/\left\{72\times \mathrm{SCr}\;\left(\mathrm{mg}/\mathrm{dL}\right)\right\}\times 0.85\;\text{in women} \end{array}$$

This value was used as crude eCCr. For the adjusted eCCr value, the modified Cockcroft–Gault formula was used by adding 0.2 mg/dL to the reported SCr value as follows:

$\begin{array}{c} \text{Adjusted eCCr}\;\left(\mathrm{mL}/\min\right)=\left\{\left(140-\mathrm{age}\;\left(\text{years}\right)\right)\times \text{weight}\;\right. \end{array}$ 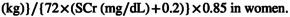


Serum creatinine has intra‐ and inter‐day fluctuations of 5%–10%^31^, ^32^, ^33^; therefore, we calculated the potential range of each eCCr by substituting the respective values obtained by multiplying the reported SCr by 1.1 or 0.9 times into each eCCr equation. Furthermore, the eCCr value in patients with obesity overestimates actual GFR.^34^ As GFR prediction accuracy is improved using adjusted ideal body weight (AIBW) instead of actual body weight (ABW) in the Cockcroft–Gault formula,^34^ we used AIBW for patients with a body mass index (BMI) of ≥30 kg/m^2^, which is defined as obese according to the World Health Organization criteria,^35^ as follows:

$$\begin{array}{c} \text{Ideal body weight}\;\left(\mathrm{IBW}\right)\;\left(\mathrm{kg}\right)=\left\{\left[\text{height}\;\left(\mathrm{cm}\right)–152.4\right]\times 0.9\right\}\\ \;+\mathrm{sex},\text{where}\;\mathrm{sex}\;\text{is}\;50\;\text{for male and}\;45.5\;\text{for female} \end{array}$$

$$\text{AIBW}\;\left(\mathrm{kg}\right)=\mathrm{IBW}+\left\{0.4\times \left[\mathrm{ABW}\;\left(\mathrm{kg}\right)–\mathrm{IBW}\;\left(\mathrm{kg}\right)\right]\right\}$$

Patients were divided into crude and adjusted groups according to the above‐described ranges. In clinical practice, truncated‐ or rounded‐off dose of chemotherapeutic agent is often used, which is potentially associated with differences between calculated and administered doses. We also assumed these differences were within this range. If patients were classified into both groups or not classified into either group, they were considered unclassified.

The accuracy of the estimation was evaluated using the mean absolute error (MAE), its standard error (MAE ± standard error), and root mean squared error (RMSE), as follows:^36^

$$\mathrm{MAE}\;\left(\mathrm{mL}/\min\right)=1/n\times \sum |\text{eCCr}-\text{bGFR}|$$

$$\mathrm{MAE}\;\left(\%\right)=1/n\times \sum \left\{\left(\text{eCCr−bGFR}\right)/\text{bGFR}\times 100\right\}$$

$$\text{RMSE}\;\left(\mathrm{mL}/\min\right)={\left\{1/n\times \sum {\left(\text{eCCr−bGFR}\right)}^{2}\right\}}^{1/2}$$

$$\text{RMSE}\;\left(\%\right)={\left\{1/n\times \sum {\left[\left(\text{eCCr−bGFR}\right)/\text{bGFR}\times 100\right]}^{2}\right\}}^{1/2}$$

### 2.5 Estimation of carboplatin AUC using Japanese estimated glomerular filtration rate

Because of the nature of primary study, the measured GFR values were not provided; therefore, to evaluate the impact of adjustment on carboplatin AUC, we used the estimated glomerular filtration rate (eGFR)^9^ instead of the measured GFR value for the estimation of AUC, as follows:

$$\begin{array}{c} \text{Estimated carboplatin AUC}\;=\;\text{actual carboplatin dose}\;\left(\mathrm{mg}\right)\\ /(\text{eGFR}\;(\mathrm{mL}/\min)\;+\;25) \end{array}$$

### 2.6 Statistical analysis

Mann–Whitney U and Fisher's exact tests were used to evaluate binary and continuous variables, respectively. Kaplan–Meier estimates were employed for time‐to‐event endpoints, including PFS and OS. Cox proportional hazards models adjusted for age (non‐older patients, age < 75 years; older patients, age ≥ 75 years), sex (male or female), performance status (PS) (0 or 1/2), smoking history (never or current/ex), clinical stage (III/IV or recurrence), epidermal growth factor receptor (*EGFR*) mutation/anaplastic lymphoma kinase (*ALK*) translocation (negative/unknown or positive), and initial AUC (<6 or 6) were used to estimate hazard ratios (HRs) and test for differences in PFS and OS between groups. In the exploratory analysis, we divided patients into non‐older (age < 75 years) and older adult (age ≥ 75 years) subpopulations and developed propensity scores using the same factors except for age in each subpopulation. Cox analyses adjusted for this propensity score were also conducted for each subpopulation between groups. Logistic regression analysis for each adverse event adjusted by group (adjusted or crude) and initial AUC (<6 or 6) were used. A two‐sided *p*‐value of <0.05 indicated a statistically significant difference. SPSS version 27 (IBM) was used for statistical analyses.

## 3 RESULTS

### 3.1 Patient characteristics

**FIGURE 1:**
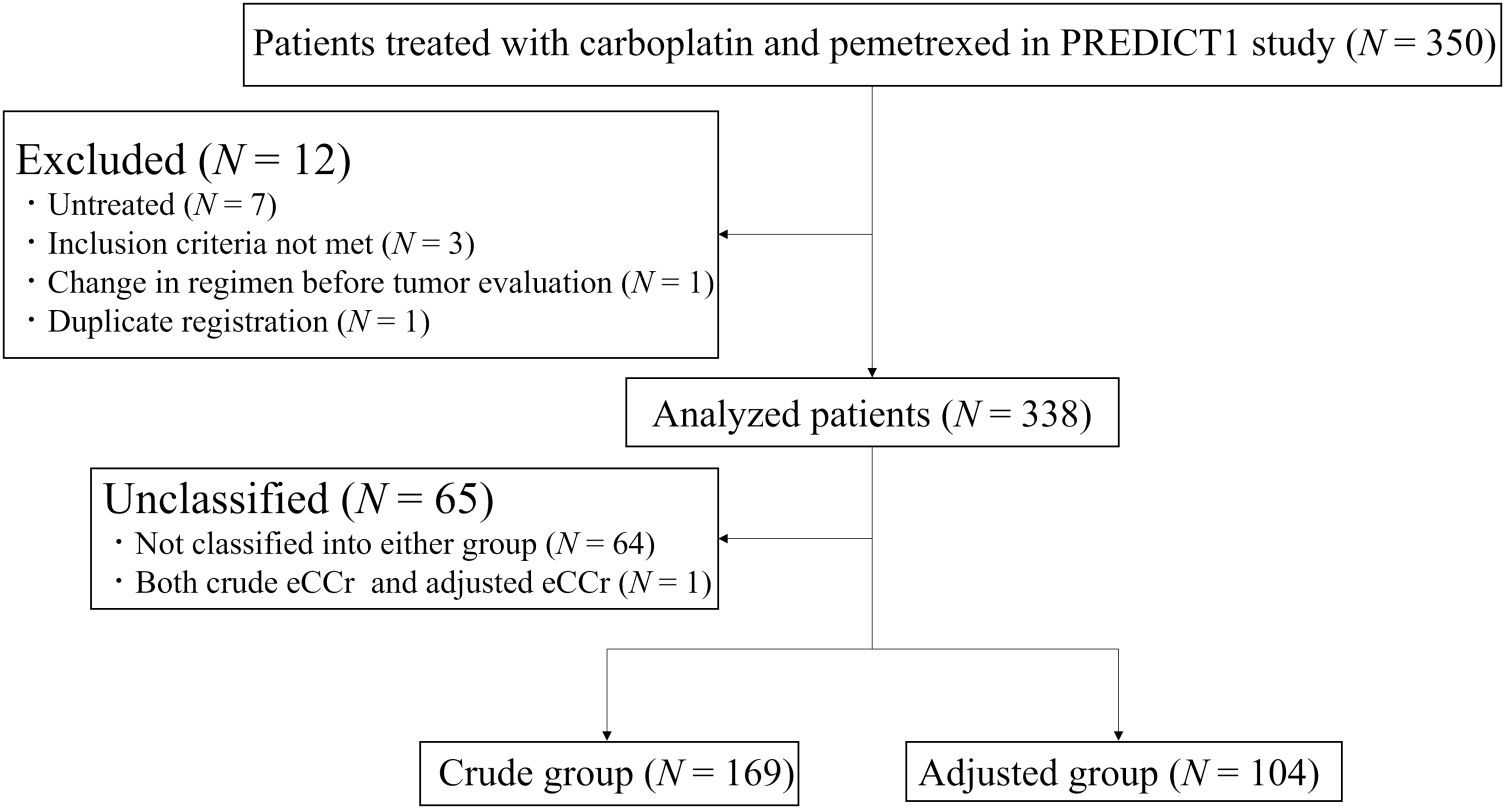
Flowchart of the study design and patients. eCCr, estimated creatinine clearance.

Total of 350 chemotherapy‐naïve patients with non‐squamous NSCLC receiving carboplatin–pemetrexed as the first‐line treatment were enrolled at 27 institutions in Japan between July 2012 and June 2017 (Figure 1). Among them, 12 patients were excluded for the following reasons: untreated (*N* = 7), inclusion criteria not met (*N* = 3), change in regimen before tumor evaluation (*N* = 1), and duplicate registration (*N* = 1). We estimated SCr adjustment in 338 patients and divided them into two groups: 169 and 104 in the crude and adjusted groups, respectively (Figure 1). Sixty‐five patients were not classified into these groups for the following reasons: above the upper limit of crude eCCr range (*N* = 17), below the lower limit of crude eCCr range, and above the upper limit of adjusted eCCr range (*N* = 30), below the lower limit of adjusted eCCr range (*N* = 17), and classified into both groups (*N* = 1). A comparison between bGFR and eCCr levels in each group is shown in Figure S1. In the crude group, the MAE and RMSE of crude eCCr and bGFR were 2.96% and 4.08%, respectively. In the adjusted group, the MAE and RMSE between adjusted eCCr and bGFR were 2.40% and 3.16%, respectively.

Table 1 lists the baseline patient characteristics. The median age in the crude group was significantly higher than that in the adjusted group (71 vs. 69 years, *p* = 0.01). The proportion of older people aged ≥75 years in the crude group was also significantly higher than that in the adjusted group (27.8% vs. 16.3%, *p* = 0.039). A BMI of ≥30 kg/m^2^ was observed in one and two patients in the crude and adjusted groups, respectively, and AIBW was used to estimate CCr in these patients. Only one patient in the crude group had a bGFR >125 mL/min. Crude eCCr values were significantly different between both groups (71.2 vs. 79.0, *p* = 0.018). The proportion of patients treated with carboplatin with a target AUC of six was significantly higher in the adjusted group (58.7%) than that in the crude group (41.4%) (*p* = 0.01). We calculated the percentages of carboplatin dosing in the adjusted group, and the median proportion based on the dose calculated using crude eCCr value was 82.7% (range, 69.1–92.6%). No significant differences in the other clinical characteristics were observed between the groups.

**TABLE 1:** Patient characteristics.

Characteristic, *N* (%)	Crude group (*N* = 169)	Adjusted group (*N* = 104)	*p*‐value^a^
Age (years)			
Median (range)	71 (44–81)	69 (40–80)	0.010
≥75	47 (27.8)	17 (16.3)	0.039
Sex			
Male	140 (82.8)	83 (79.8)	0.525
Female	29 (17.2)	21 (20.2)	
Smoking history			
Current or ex‐smoker	143 (84.6)	88 (84.6)	1.000
Never smoker	26 (15.4)	16 (15.4)	
ECOG PS			
0	86 (50.9)	40 (38.5)	0.121
1	73 (43.2)	57 (54.8)	
2	10 (5.9)	7 (6.7)	
BSA (m^2^)			
Median (range)	1.62 (1.24–2.06)	1.62 (1.21–2.16)	0.227
BMI (kg/m^2^)			
Median (range)	21.6 (14.7–30.6)	22.4 (15.9–32.1)	0.095
<18.5	26 (15.4)	13 (12.5)	
18.5≤ and <25	114 (67.5)	70 (67.3)	
25≤ and <30	28 (16.6)	19 (18.3)	
≥30	1 (0.6)	2 (1.9)	
Histology			
Adenocarcinoma	159 (94.1)	97 (93.3)	0.801
Others	10 (5.9)	7 (6.7)	
Stage			
III	24 (14.2)	14 (13.5)	0.805
IV	134 (79.3)	81 (77.9)	
Postoperative recurrence	11 (6.5)	9 (8.7)	
*EGFR* mutation or *ALK* fusion gene			
Positive	16 (9.5)	16 (15.4)	0.175
Negative or unknown	153 (90.5)	88 (84.6)	
Serum creatinine (mg/dL)			
Median (range)	0.76 (0.38–1.38)	0.72 (0.34–1.38)	0.244
Male: median (range)	0.79 (0.43–1.38)	0.75 (0.42–1.38)	0.286
Female: median (range)	0.54 (0.38–0.80)	0.58 (0.34–0.76)	0.813
eGFR^b^ (mL/min)			
Median (range)	70.7 (39.1–127.2)	75.6 (35.0–139.8)	0.077
Crude eCCr (mL/min)			
Median (range)	71.2 (40.1–132.1)	79.0 (36.0–170.8)	0.018
<45	1 (0.6)	5 (4.8)	0.031
Adjusted eCCr (mL/min)			
Median (range)	56.0 (34.5–100.1)	60.3 (31.4–122.0)	0.024
bGFR (mL/min)			
Median (range)	71.0 (38.3–132.0)	61.4 (29.0–120.0)	<0.001
>125	1 (0.6)	0	
Target AUC			
6	70 (41.4)	61 (58.7)	0.010
5.4	1 (0.6)	0	
5	98 (58.0)	43 (41.3)	

Abbreviations: ALK, anaplastic lymphoma kinase; AUC, area under the blood concentration‐time curve; bGFR, back‐calculated glomerular filtration rate; BMI, body mass index; BSA, body surface area; eCCr, estimated creatine clearance; ECOG PS, Eastern Cooperative Oncology Group performance status; EGFR, epidermal growth factor receptor; eGFR, estimated glomerular filtration rate.

^a^ *p*‐values were calculated using the Mann–Whitney *U* test or Fisher's exact test.

^b^ eGFR was calculated using the following formula:^9^ eGFR (mL/min/1.73 m^2^) = 194 × age (years)^(−0.287)^ × serum creatinine (mg/dL)^(−1.094)^ × 0.7939 (in women). eGFR (mL/min) = eGFR (mL/min/1.73 m^2^) × height (cm)^(0.725)^ × actual body weight (kg)^(0.425)^ × 0.007184/1.73.

Among 64 patients aged ≥75 years, the baseline characteristics were similar between groups, except for PS (PS = 0, 48.9% vs. 29.4%) (Table 2). The proportion of patients with each target AUC was similar in both groups among older adults.

**TABLE 2:** Characteristics of older and non‐older patients.

	Older patients		Non‐older patients	
Characteristic, *N* (%)	Crude group (*N* = 47)	Adjusted group (*N* = 17)	Crude group (*N* = 122)	Adjusted group (*N* = 87)
Age (years)				
Median (range)	77 (75–81)	76 (75–80)	68 (44–74)	67 (40–74)
Sex				
Male	38 (80.9)	14 (82.4)	102 (83.6)	69 (79.3)
Female	9 (19.1)	3 (17.6)	20 (16.4)	18 (20.7)
Smoking history				
Current or ex‐smoker	36 (76.6)	14 (82.4)	107 (87.7)	74 (85.1)
Never smoker	11 (23.4)	3 (17.6)	15 (12.3)	13 (14.9)
ECOG PS				
0	23 (48.9)	5 (29.4)	63 (51.6)	35 (40.2)
1	23 (48.9)	12 (70.6)	50 (41.0)	45 (51.7)
2	1 (2.1)	0	9 (7.4)	7 (8.0)
BSA (m^2^)				
Median (range)	1.60 (1.28–2.06)	1.57 (1.28–1.74)	1.62 (1.24–2.02)	1.64 (1.21–2.16)
BMI (kg/m^2^)				
Median (range)	22.7 (16.9–30.6)	21.0 (17.9–25.7)	21.1 (14.7–28.8)	22.4 (15.9–32.1)
<18.5	1 (2.1)	2 (11.8)	25 (20.5)	11 (12.6)
18.5≤ and <25	37 (78.7)	13 (76.5)	77 (63.1)	57 (65.5)
25≤ and <30	8 (17.0)	2 (11.8)	20 (16.4)	17 (19.5)
≥30	1 (2.1)	0	0	2 (2.3)
Histology				
Adenocarcinoma	44 (93.6)	16 (94.1)	115 (94.3)	81 (93.1)
Others	3 (6.4)	1 (5.9)	7 (5.7)	6 (6.9)
Stage				
III	9 (19.1)	1 (5.9)	15 (12.3)	13 (14.9)
IV	36 (76.6)	14 (82.4)	98 (80.3)	67 (77.0)
Postoperative recurrence	2 (4.3)	2 (11.8)	9 (7.4)	7 (8.0)
*EGFR* mutation or *ALK* fusion gene				
Positive	3 (6.4)	1 (5.9)	13 (10.6)	15 (7.2)
Negative or unknown	44 (93.6)	16 (94.1)	109 (89.3)	72 (92.8)
Serum creatinine (mg/dL)				
Median (range)	0.79 (0.47–1.10)	0.81 (0.50–1.04)	0.74 (0.38–1.38)	0.72 (0.34–1.38)
Male: median (range)	0.83 (0.58–1.10)	0.82 (0.59–1.04)	0.77 (0.43–1.38)	0.74 (0.42–1.38)
Female: median (range)	0.56 (0.47–0.71)	0.52 (0.50–0.71)	0.54 (0.38–0.80)	0.59 (0.34–0.76)
eGFR^a^ (mL/min)				
Median (range)	63.4 (49.0–93.3)	64.2 (45.9–98.0)	72.3 (39.1–127.2)	79.2 (35.0–139.8)
Crude eCCr (mL/min)				
Median (range)	61.5 (45.3–102.9)	64.5 (44.1–96.9)	74.9 (40.1–132.1)	83.4 (36.0–170.8)
<45	0	1 (5.9)	1 (0.8)	4 (4.6)
Adjusted eCCr (mL/min)				
Median (range)	48.8 (34.5–72.2)	50.2 (34.4–72.4)	59.1 (35.0–100.2)	66.1 (31.4–122.0)
bGFR (mL/min)				
Median (range)	61.0 (46.7–101.0)	50.0 (36.4–72.2)	75.0 (38.3–132.0)	65.3 (29.0–120.0)
>125	0	0	1 (0.9)	0
Target AUC				
6	17 (36.2)	7 (41.2)	53 (43.4)	54 (62.1)
5.4	0	0	1 (0.9)	0
5	30 (63.8)	10 (58.8)	68 (55.7)	33 (37.9)

Abbreviations: ALK, anaplastic lymphoma kinase; AUC, area under the blood concentration‐time curve; bGFR, back‐calculated glomerular filtration rate; BMI, body mass index; BSA, body surface area; eCCr, estimated creatinine clearance; ECOG PS, Eastern Cooperative Oncology Group performance status; EGFR, epidermal growth factor receptor; eGFR, estimated glomerular filtration rate.

^a^ eGFR was calculated using the following formula:^9^ eGFR (mL/min/1.73 m^2^) = 194 × age (years)^(−0.287)^ × serum creatinine (mg/dL)^(−1.094)^ × 0.7939 (in women). eGFR (mL/min) = eGFR (mL/min/1.73 m^2^) × height (cm)^(0.725)^ × actual body weight (kg)^(0.425)^ × 0.007184/1.73.

Dose reduction was conducted in 24 (14.2%) and 14 (13.5%) in the crude and adjusted groups, respectively. In older patients, dose reduction was conducted in 12 (25.5%) and 3 (17.6%) in the crude and adjusted groups, respectively. In non‐older patients, dose reduction was conducted in 12 (9.8%) and 11 (12.6%) in the crude and adjusted groups, respectively.

### 3.2 Efficacy

**FIGURE 2:**
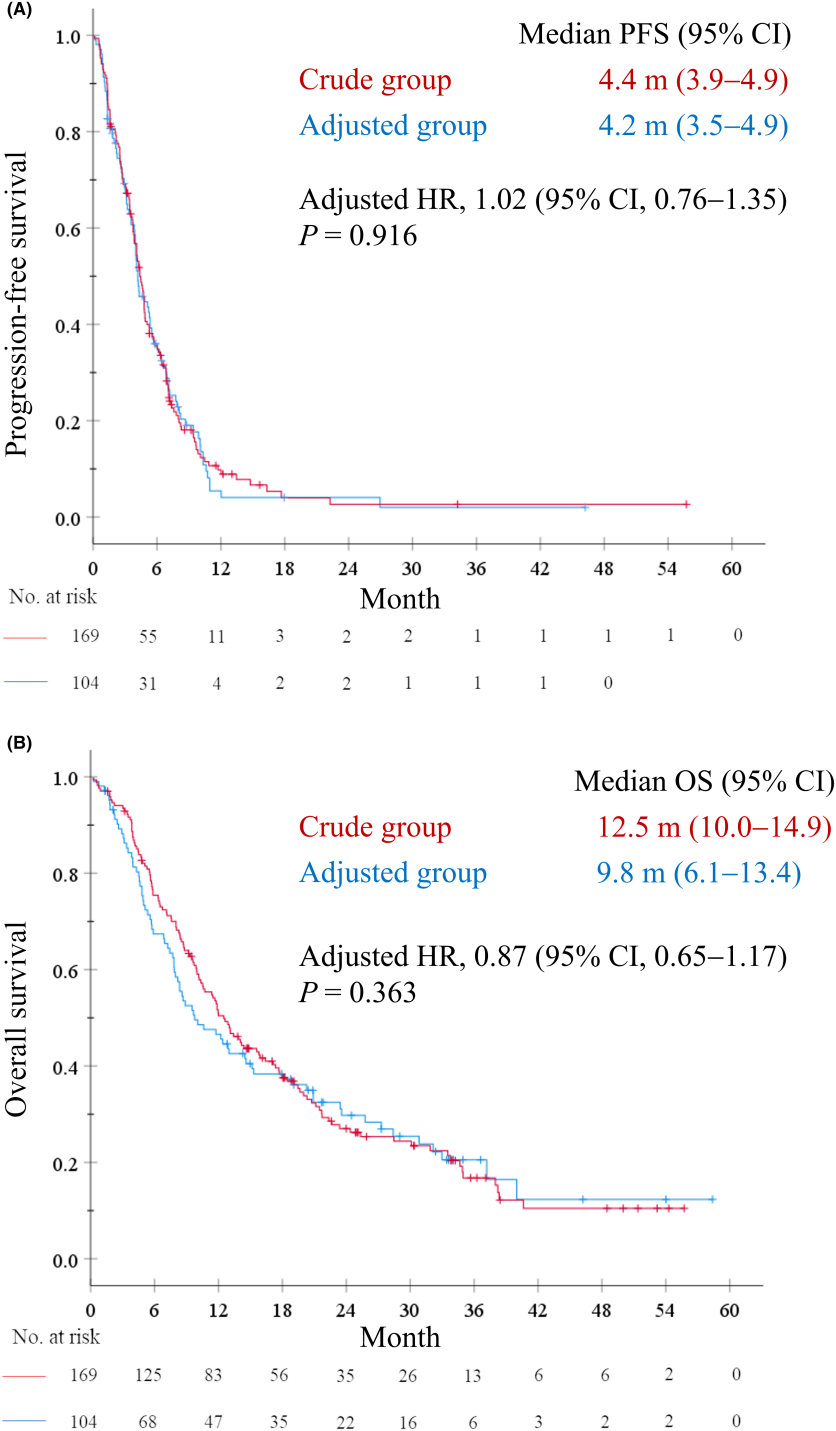
Kaplan–Meier plots for (A) progression‐free survival and (B) overall survival. CI, confidence interval; HR, hazard ratio; OS, overall survival; PFS, progression‐free survival.

Response rates (RRs) in the crude and adjusted groups were 22.5% and 26.0%, respectively (*p* = 0.559), and disease control rates were 77.9% and 81.1%, respectively (*p* = 0.537). Unclassified patients showed similar response and disease control rates, except those treated with the lower limit of the adjusted eCCr range (Figure S2). The median PFS was 4.4 months (95% confidence interval [CI], 3.9–4.9) in the crude group and 4.2 months (95% CI, 3.5–4.9) in the adjusted group. The adjusted HR for PFS was 1.02 (95% CI, 0.76–1.35; *p* = 0.916) (Figure 2A). The median OS was 12.5 months (95% CI, 10.0–14.9) in the crude group and 9.8 months (95% CI, 6.1–13.4) in the adjusted group. The adjusted HR for OS was 0.87 (95% CI, 0.65–1.17; *p* = 0.363) (Figure 2B).

**FIGURE 3:**
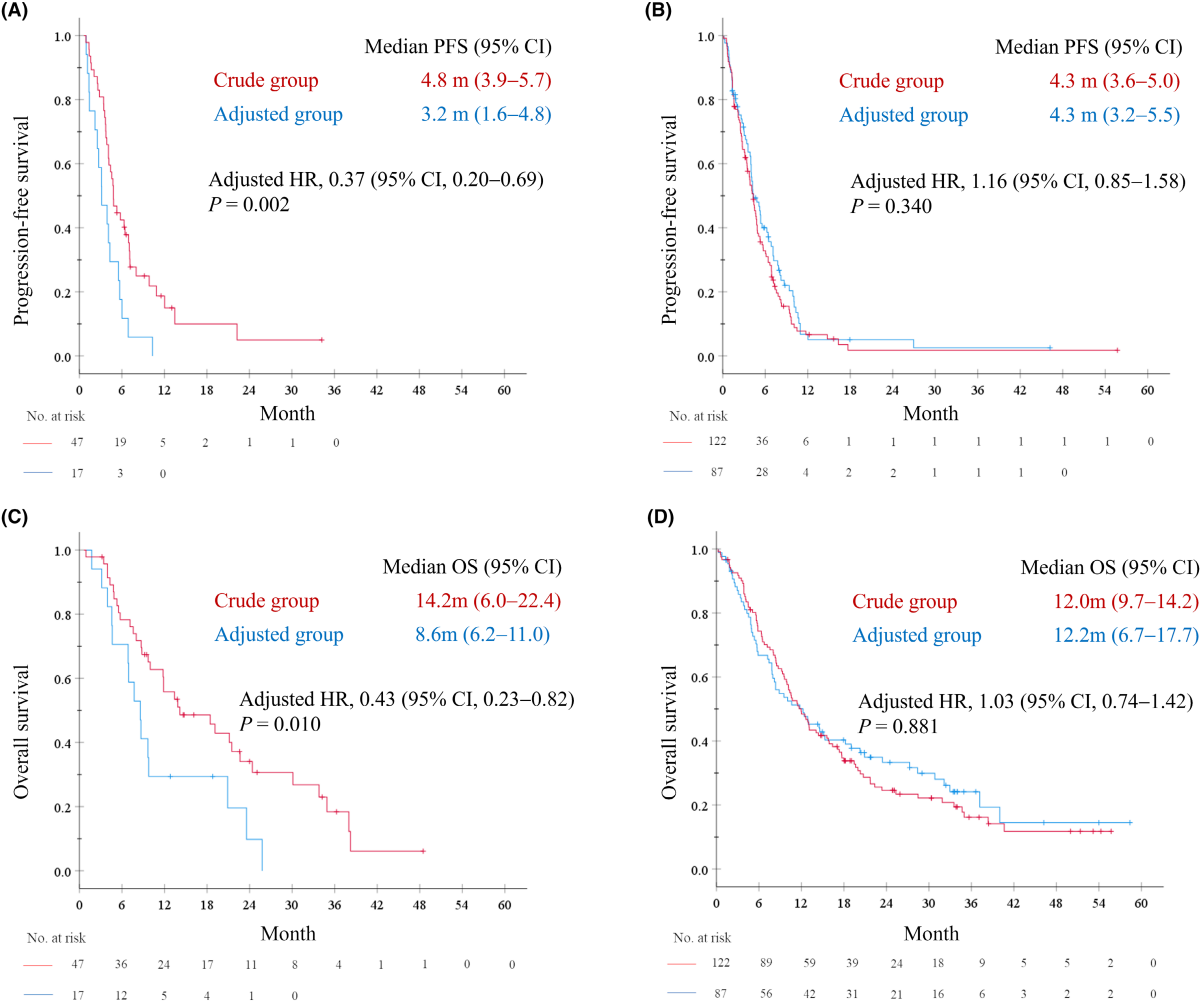
Kaplan–Meier plots for progression‐free survival (A), (B) and overall survival (C), (D) in older (age ≥ 75) and non‐older patients (age <75), respectively. CI, confidence interval; HR, hazard ratio; OS, overall survival; PFS, progression‐free survival.

Among the 64 patients aged ≥75 years, RR in crude and adjusted groups were 27.7% and 17.6%, respectively (*p* = 0.525). The median PFS was 4.8 months (95% CI, 3.9–5.7) in the crude group (*N* = 47) and 3.2 months (95% CI, 1.6–4.8) in the adjusted group (*N* = 17; Figure 3A). Additionally, among older patients, the median OS was 14.2 months (95% CI, 6.0–22.4) in the crude group and 8.6 months (95% CI, 6.2–11.0) in the adjusted group (Figure 3C), whereas similar PFS and OS were observed in non‐older patients. The median PFS was 4.3 months in both the crude (*N* = 122; 95% CI, 3.6–5.0) and adjusted groups (*N* = 87; 95% CI, 3.2–5.5; Figure 3B). In non‐older patients, the median OS was 12.0 months (95% CI, 9.7–14.2) in the crude group and 12.2 months (95% CI, 6.7–17.7) in the adjusted group (Figure 3D). In older patients, Cox proportional hazards models adjusted by propensity score calculated using sex, PS, smoking history, clinical stage, EGFR/ALK, and initial AUC revealed that the adjusted HR for PFS and OS was 0.37 (95% CI, 0.20–0.69; *p* = 0.002) and 0.43 (95% CI, 0.23–0.82; *p* = 0.010), respectively, between both groups. Conversely, in non‐older patients, the adjusted HR for PFS and OS were 1.16 (95% CI, 0.85–1.58; *p* = 0.340) and 1.03 (95% CI, 0.74–1.42; *p* = 0.881), respectively, between groups.

We conducted additional analyses to evaluate the impact of adjustment on carboplatin AUC. We used eGFR^9^ instead of measured GFR value for the estimation of AUC, and evaluated the relationship between estimated carboplatin AUC and RR for all patients (*N* = 338) (Figure S3). As estimated carboplatin AUC increased to >5, there seemed to be no remarkable increase in RR. In addition, based on these data, AUC >4.25 calculated using eGFR seemed to be at least needed for response of 20% (Figure S3). In the adjusted group, the median AUC was 4.96, and 17 of 104 patients (16.3%) had AUC <4.25 (Figure S4A). Regarding non‐older patients in the adjusted group, 10 of 87 patients (11.5%) had AUC below this value (Figure S4C); meanwhile, 7 of 17 older patients (41.2%) had AUC below this value (*p* = 0.0067) (Figure S4B).

For subsequent therapy, 106 of 169 patients (62.7%) in the crude group and 65 of 104 patients (62.5%) in the adjusted group received at least one therapy (*p* = 1.000). Antibodies against programmed cell death‐1 or its ligand were administered to 33 patients (19.5%) in the crude group and 19 patients (18.3%) in the adjusted group (*p* = 0.578). EGFR or ALK tyrosine kinase inhibitors were administered to 20 patients (11.8%) in the crude group and 15 patients (14.4%) in the adjusted group (*p* = 0.874).

### 3.3 Safety

Frequency of hematologic toxicity was generally higher in the crude group than in the adjusted group (Table 3). The incidence of Grade 3 or 4 neutropenia was significantly higher in the crude group than that in the adjusted group (66 [39.1%] vs. 28 [26.9%]; *p* = 0.049). Similarly, crude eCCr was associated with a higher incidence of Grade 3 or 4 anemia and thrombocytopenia than that with adjusted eCCr (anemia: 53 [31.4%] vs. 24 [23.1%], *p* = 0.166; thrombocytopenia: 62 [36.7%] vs. 29 [27.9%], *p* = 0.147). The incidence of Grade 3 or 4 non‐hematologic toxicity was similar in both groups. We conducted a logistic regression analysis adjusted by initial AUC to evaluate the odds ratio between both groups, which showed a significantly higher incidence of Grade 3 or 4 neutropenia in the crude group, with an adjusted odds ratio of 1.91 (95% CI, 1.11–3.3, *p* = 0.02) (Table S1). Treatment‐related deaths occurred due to pneumonia in 1 of 169 patients (0.6%) in the crude group and pneumonitis in 3 of 104 patients (2.9%) in the adjusted group.

**TABLE 3:** Treatment‐related adverse events.

Adverse event, *N* (%)	Crude group (*N* = 169)		Adjusted group (*N* = 104)		*p*‐value^a^
	Any grade	Grade 3 or 4	Any grade	Grade 3 or 4	Grade 3 or 4
Neutrophil count decreased	119 (70.4)	66 (39.1)	69 (66.3)	28 (26.9)	0.049
Anemia	159 (94.1)	53 (31.4)	98 (94.2)	24 (23.1)	0.166
Platelet count decreased	128 (75.7)	62 (36.7)	86 (82.7)	29 (27.9)	0.147
FN	9 (5.3)	9 (5.3)	6 (5.8)	6 (5.8)	1.000
AST or ALT increased	100 (59.2)	7 (4.1)	65 (62.5)	4 (3.8)	1.000
Creatinine increased	46 (27.2)	0	35 (33.7)	0	NA
Nausea or vomiting	104 (61.5)	9 (5.3)	78 (75.0)	5 (4.8)	1.000
Diarrhea	18 (10.7)	2 (1.2)	17 (16.3)	0	0.527
Constipation	114 (67.5)	9 (5.3)	79 (76.0)	6 (5.8)	1.000
Fatigue	120 (71.0)	21 (12.4)	77 (74.0)	9 (8.7)	0.426
Anorexia	125 (74.0)	19 (11.2)	87 (83.7)	14 (13.5)	0.573

Abbreviations: ALT, alanine aminotransferase; AST, aspartate aminotransferase; FN, febrile neutropenia; NA, not applicable.

^a^ *p*‐values were calculated using Fisher's exact test.

Among the 64 patients aged ≥75 years, the frequency of Grade 3 or 4 hematologic toxicity in the crude group was similar to that in non‐older patients, whereas older patients in the adjusted group had a lower incidence of Grade 3 or 4 hematologic toxicity than that in the non‐older group (Table S2). Grade 3 or 4 neutropenia, anemia, and thrombocytopenia were observed in 3 (17.6%), 2 (11.8%), and 3 (17.6%) patients in the older adjusted group, respectively, compared with 25 (28.7%), 22 (25.3%), and 26 (29.9%) patients in non‐older adjusted group, respectively.

## 4 DISCUSSION

In this post hoc analysis of a multicenter prospective observational trial of carboplatin–pemetrexed treatment in patients with non‐squamous NSCLC, patients were divided into two groups (adjusted and crude groups) by comparing bGFR based on both actual administered carboplatin dosage and reported AUC with eCCr. Similar clinical efficacy was demonstrated in terms of RR (22.5% and 26.0% in the crude and adjusted groups, respectively, *p* = 0.559), PFS (adjusted HR, 1.02; 95% CI, 0.76–1.35), and OS (adjusted HR, 0.87; 95% CI, 0.65–1.17) between both the groups. Regarding toxicity, patients in the adjusted group tended to have a lower incidence of hematologic adverse events than those in the crude group. However, although the number of cases was small, patients aged ≥75 years in the adjusted group had significantly shorter PFS and OS with a considerably lower incidence of hematologic toxicity than those in the crude group, even after propensity score adjustment of patient background (adjusted HRs for PFS and OS were 0.37 [95% CI, 0.20–0.69; *p* = 0.002] and 0.43 [95% CI, 0.23–0.82; *p* = 0.010], respectively). These results indicate that adjustment of SCr measured using the enzymatic method in CCr calculation using the Cockcroft–Gault formula is associated with similar clinical efficacy and a low incidence of toxicity compared with those of non‐adjusted values in patients with preserved renal function eligible for carboplatin–pemetrexed treatment, although should be performed with special caution when administered to older patients.

In cytotoxic chemotherapy, the dose–response curve for most drugs eventually plateaus, and toxicity increases with an increase in chemotherapy dose.^37^ In a previous study of ovarian cancer, increasing carboplatin AUC above 5–7 did not improve the likelihood of response, yet increased myelotoxicity.^4^ In the current study, similar to this study, as estimated carboplatin AUC increased to >5, there seemed to be no remarkable increase in RR (Figure S3). Based on these data, AUC >4.25 calculated using eGFR seemed to be at least needed for response of 20%. In the adjusted group, median AUC was 4.96, and only 16.3% of patients had AUC <4.25, which was potentially associated with similar clinical efficacy in the patients in the adjusted group compared with those in the crude group (Figure S4). Furthermore, although the crude eCCr value was approximately 30% higher than the adjusted eCCr value (Figure S1), the RR in the crude group was not superior to that in the adjusted group (*p* = 0.559). Moreover, the frequency of hematologic toxicity was generally higher in the crude group than in the adjusted group (Table 3). Some clinical guidelines have recommended capping the carboplatin dose to avoid potential adverse events due to overdosing.^38^, ^39^ The maximum dose is based on an estimated GFR capped at 125 mL/min in patients with normal renal function. Although some patients exhibited high crude eCCr values, the adjusted eCCr value did not exceed 125 mL/min, indicating that SCr adjustment plays a role similar to capping in terms of avoiding overdosing. These results are in line with those of previous studies on ovarian cancer.

In previous prospective studies, SCr adjustment was used for carboplatin dosing. Kim et al.^40^ and Minami et al.^41^ conducted a Phase II trial of carboplatin–pemetrexed followed by maintenance pemetrexed treatment in patients with advanced non‐squamous NSCLC in a similar manner. The actual carboplatin dose (target AUC = 6) was calculated based on Cockcroft–Gault and Calvert's formula, while the adjusted eCCr was used to substitute GFR in Calvert's formula. The authors reported an RR, median PFS, and OS of 32.4%–51%, 5.2–6.3 months, and 23.3–24.3 months, respectively. Grade 3 or 4 neutropenia and thrombocytopenia were observed in 33%–41% and 18%–29% of patients, respectively. Okamoto et al.^42^ also conducted a Japanese study of carboplatin–pemetrexed with maintenance pemetrexed in a similar manner, except for carboplatin dosing, which was not adjusted despite SCr being measured using the enzymatic method. The authors reported similar findings to those of Kim et al.^40^ and Minami et al.^41^ in terms of efficacy, with RR, median PFS, and OS of 35.8%, 5.7 months, and 20.2 months, respectively; however, the incidence of hematologic toxicities was higher than that in those studies. Grade 3–4 neutropenia and thrombocytopenia occurred in 56.9% and 41.3% of patients, respectively. These differences are probably attributable to the difference in SCr adjustment levels and are consistent with our findings.

In the exploratory analysis for older patients, PFS and OS in the crude group were longer than those in the adjusted group, even after propensity score adjustment of patients' background, including PS, clinical stage, and target AUC, which showed differences between both the groups (Figure 3). The frequency of hematologic toxicity in the adjusted group was considerably lower than that of previous studies^26^, ^40^, ^41^ and that of the non‐older adjusted group (Table S2), indicating that SCr adjustment was associated with underestimation of the true GFR in those populations. Although only 11.5% of non‐older patients in the adjusted group exhibited the estimated carboplatin AUC <4.25, which seemed to be the lowest value needed for a response of 20%, the AUC of 41.2% of older patients were below this value (*p* = 0.0067) (Figure S4), which was potentially associated with reduced efficacy in older patients in the adjusted group. The Cockcroft–Gault formula was developed in a relatively large population (*N* = 249); however, the majority were aged <65 years and enrolled patients aged >70 years (*N* = 59) had poor renal function with a mean CCr of 38 mL/min^5^ which may be associated with underestimation by this equation in older patients with preserved renal function. Indeed, the Cockcroft–Gault formula is approximately 30 mL/min lower than the reference estimation using inulin clearance in healthy older people, although not in young people.^27^ Additionally, a previous study comparing the Cockcroft–Gault formula using different SCr measurements with ^51^Cr‐EDTA clearance in older patients (mean age, 80 years) with mild to moderate kidney disease reported that eCCr calculated using creatinine measured using the Jaffé method tended to underestimate GFR; however, eCCr estimated using one of the enzymatic methods produced the highest GFR estimate compared with the reference ^51^Cr‐EDTA clearance.^28^ Moreover, the Cockcroft–Gault formula is based on SCr and 24‐h creatinine excretion per body weight, which decreases with age. Regarding Japanese participants, such decrease is smaller compared with non‐Asian participants, which may also be associated with underestimation in older patients.^43^ Altogether, SCr adjustment, which was calibrated to that of the non‐adjusted Jaffé's method, may be associated with GFR underestimation in older patients with a preserved renal function who are potential candidates for pemetrexed treatment, resulting in possibly low efficacy of chemotherapy and considerably low hematologic toxicity. Further studies are warranted to clarify the optimal method for GFR estimation for carboplatin dosing, especially in older adults.

Chemotherapy combined with immunotherapy has become the standard therapy for metastatic NSCLC.^6^, ^44^, ^45^, ^46^, ^47^ In these studies, crude eCCr calculated using the Cockcroft–Gault formulas was used as a substitute for GFR in Calvert's formulas for carboplatin dosing in their protocol.^6^, ^44^ Meanwhile, cytotoxic chemotherapy was used herein for cytotoxic effects and enhanced modulation of the immune response through programmed cell death‐1 or its ligand inhibition.^6^ Therefore, the effect of SCr adjustment on the clinical efficacy of these regimens remains unclear, and further studies are necessary.

In the current study, patients with crude eCCr <45 mL/min were included, which may not be suitable for carboplatin plus pemetrexed treatment (Table 1); however, in a clinical practice, those patients are potentially treated with this combination. Indeed, several retrospective studies included patients with CCr < 45 mL/min.^48^, ^49^, ^50^ In the comparison between the adjusted and crude groups, the proportion of patients with crude eCCr <45 mL/min was small but statistically greater in the adjusted group (0.6% and 4.8% in the crude and adjusted groups, respectively. *p* = 0.031). The frequency of hematologic toxicities in the crude group was generally higher than that in the adjusted group (Table S2), indicating adjustment had more impact on toxicities. When we excluded patients with crude eCCr <45 mL/min, the efficacy in terms of RR, PFS, and OS was similar to those included (data not shown). Therefore, despite having a potential effect on the efficacy and toxicity of the combination therapy, the effect on the results seemed to be limited.

This study has some limitations. First, since the study was a post hoc analysis, the actual GFR and AUC of carboplatin were not measured. Furthermore, a detailed method for estimating GFR values remains lacking. In this study, patients were divided into two groups by comparing bGFR back‐calculated from the actual administered carboplatin dosage and reported AUC with eCCr, and these exhibited a small range of MAE and RMSE (Figure S1), indicating the accuracy of estimation for SCr adjustment. Second, both the target AUC (5–6) and the method for GFR estimation were at the investigator's discretion. This may be associated with selection bias among groups. Third, in the analysis of older patients, SCr adjustment in the Cockcroft–Gault formula might be associated with low chemotherapy efficacy; however, this finding is inconclusive due to the limited number of patients. Our results remain to be confirmed in a randomized prospective study or a larger multicenter observational study for real‐world evidence.

Adjustment of SCr measured using the enzymatic method in GFR estimation with the Cockcroft–Gault formula for carboplatin dosage in patients with NSCLC with preserved renal function may be associated with similar efficacy and low toxicity compared with those of the crude SCr value. However, adjustments should be used with special caution in older patients owing to the potential for reduced efficacy.

## AUTHOR CONTRIBUTIONS

**Takahiro Hatta:** Conceptualization (lead); formal analysis (lead); writing – original draft (equal); writing – review and editing (supporting). **Tetsunari Hase:** Conceptualization (lead); formal analysis (lead); writing – original draft (lead); writing – review and editing (lead). **Toru Hara:** Data curation (equal). **Tomoki Kimura:** Data curation (equal). **Eiji Kojima:** Data curation (equal). **Takashi Abe:** Data curation (equal). **Yoshitsugu Horio:** Data curation (equal). **Yasuhiro Goto:** Data curation (equal). **Naoya Ozawa:** Investigation (equal). **Naoyuki Yogo:** Investigation (equal). **Hirofumi Shibata:** Investigation (equal). **Tomoya Shimokata:** Investigation (equal). **Tetsuya Oguri:** Data curation (equal). **Masashi Yamamoto:** Data curation (equal). **Kiyoshi Yanagisawa:** Funding acquisition (lead). **Masahiko Ando:** Formal analysis (equal). **Yuichi Ando:** Investigation (equal). **Masashi Kondo:** Supervision (equal). **Makoto Ishii:** Supervision (equal). **Yoshinori Hasegawa:** Supervision (equal).

## FUNDING INFORMATION

This work was supported by Japan Agency for Medical Research and Development (AMED) (grant number 16cm0106408h0001 [to KY]).

## CONFLICT OF INTEREST STATEMENT

T. Hase received research funding from AstraZeneca, Chugai Pharmaceutical Co., Ltd., and Novartis Pharma. K.K. Y. Ando received personal fees from Chugai Pharmaceutical Co. and research funding from Novartis Pharma K.K., Ono Pharmaceutical Co., Ltd., Yakult Honsha Co., Ltd., Chugai Pharmaceutical Co., Ltd., BeiGene Inc., and Geo Holdings Co., Ltd. Y. Hasegawa received personal fees from Novartis Pharma K.K., Boehringer Ingelheim, and AstraZeneca. The grant was paid to each institution. All remaining authors have no conflicts of interest to declare.

## ETHICS APPROVAL

The primary study protocol^29^ was approved by the institutional ethics committee of each participating institution. This post hoc analysis was approved by the Ethics Review Committee of Nagoya University Graduate School of Medicine (No. 2018–0386). Written informed consent was obtained from all patients in the primary study. The primary study was registered in UMIN Clinical Trials Registry (UMIN000008476). Animal Studies: N/A.

## PATIENT CONSENT

Written informed consent was obtained from all patients.

## Supporting information


Figure S1.

Figure S2.

Figure S3.

Figure S4.

Table S1.

Legends

## Data Availability

All data included in this study are available upon reasonable request from the corresponding author.
