# Supplementary figures and images for: Adjustment of creatinine clearance for carboplatin dosing in Calvert's formula and clinical efficacy for lung cancer
Source: Cancer Med. 2023 Jun 23;12(15):15955–69. doi: 10.1002/cam4.6235 (PMC10469651; doi:10.1002/cam4.6235)

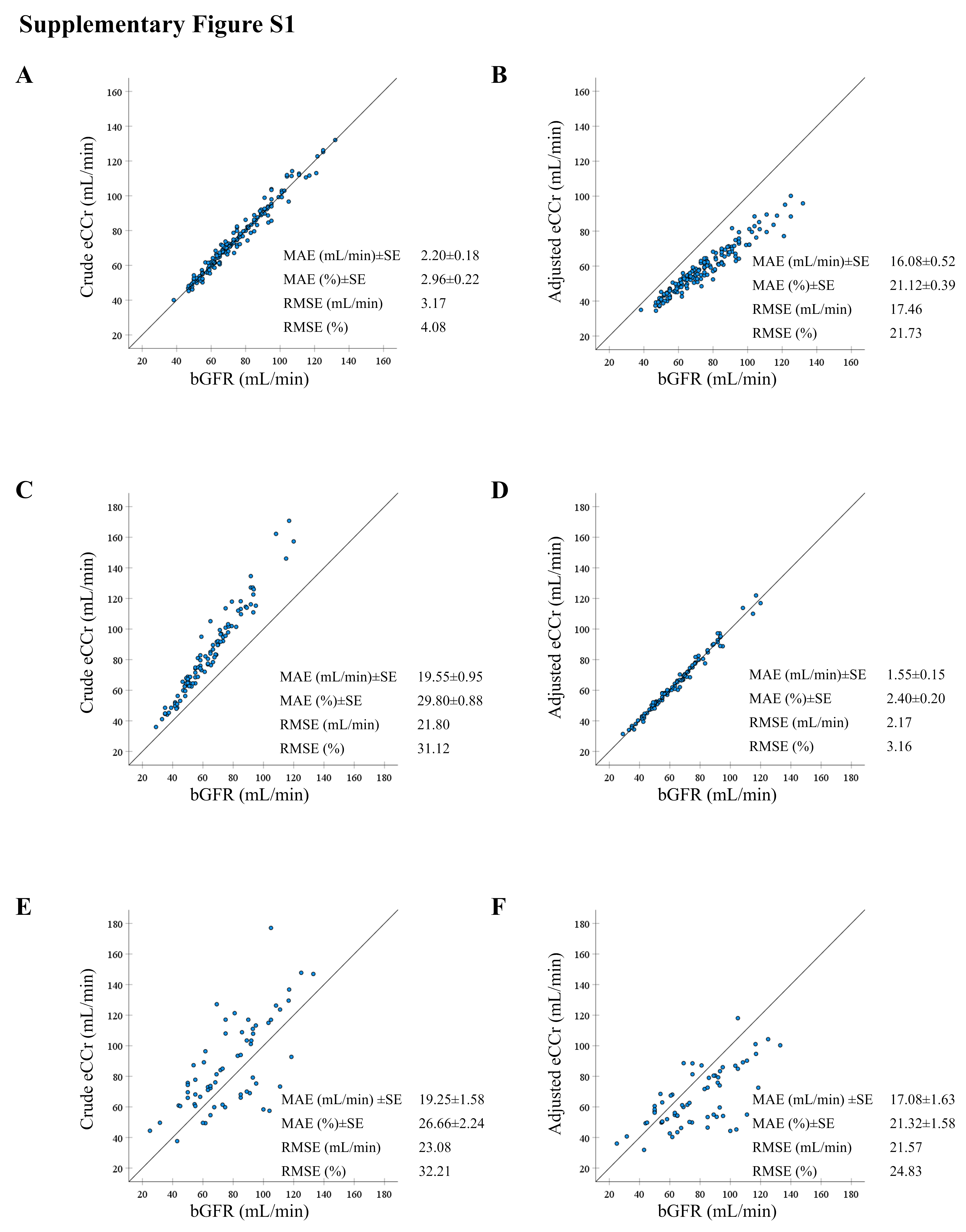

Supplement: Supplementary file 1 — Figure S1. [file CAM4-12-15955-s001.tif]

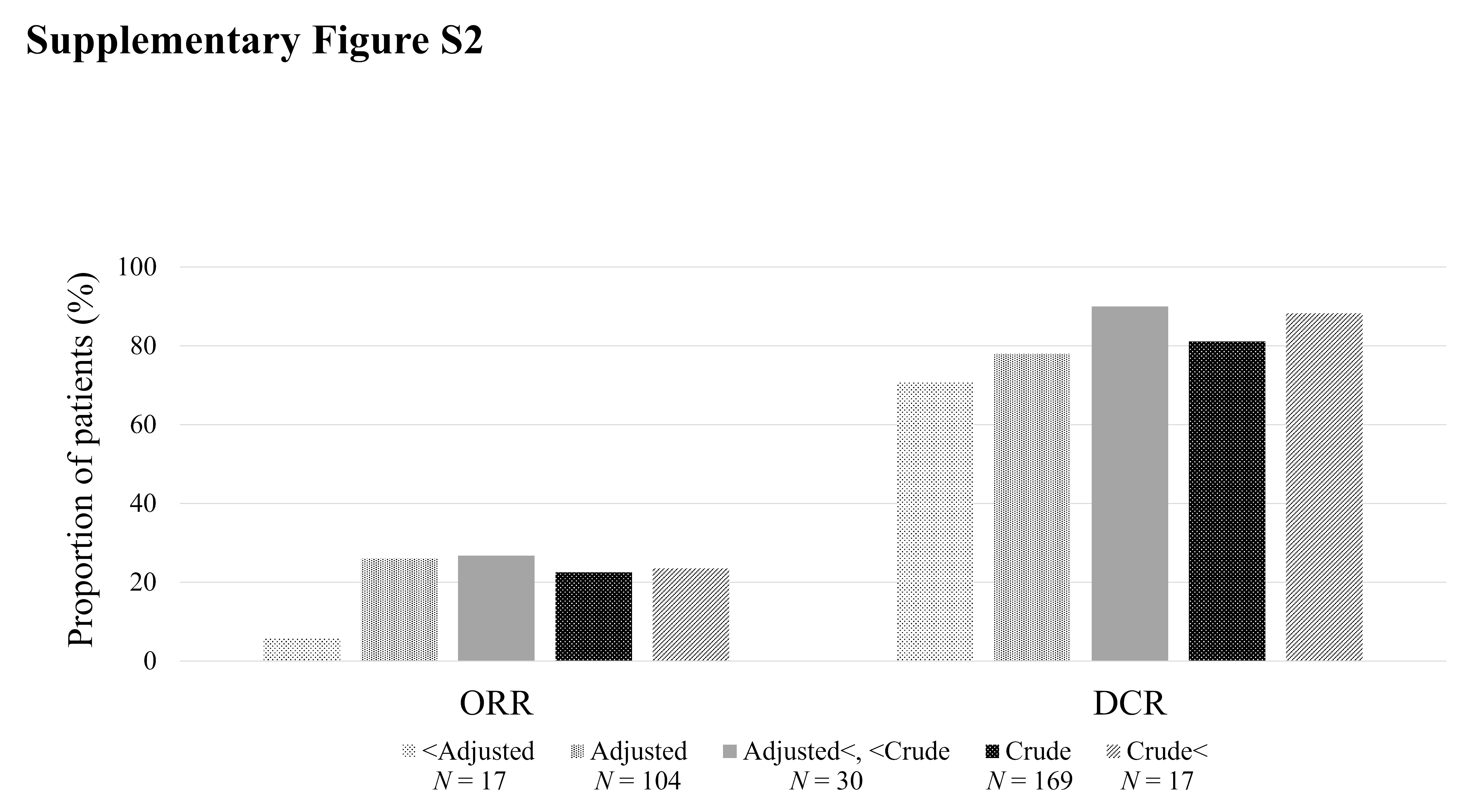

Supplement: Supplementary file 2 — Figure S2. [file CAM4-12-15955-s002.tif]

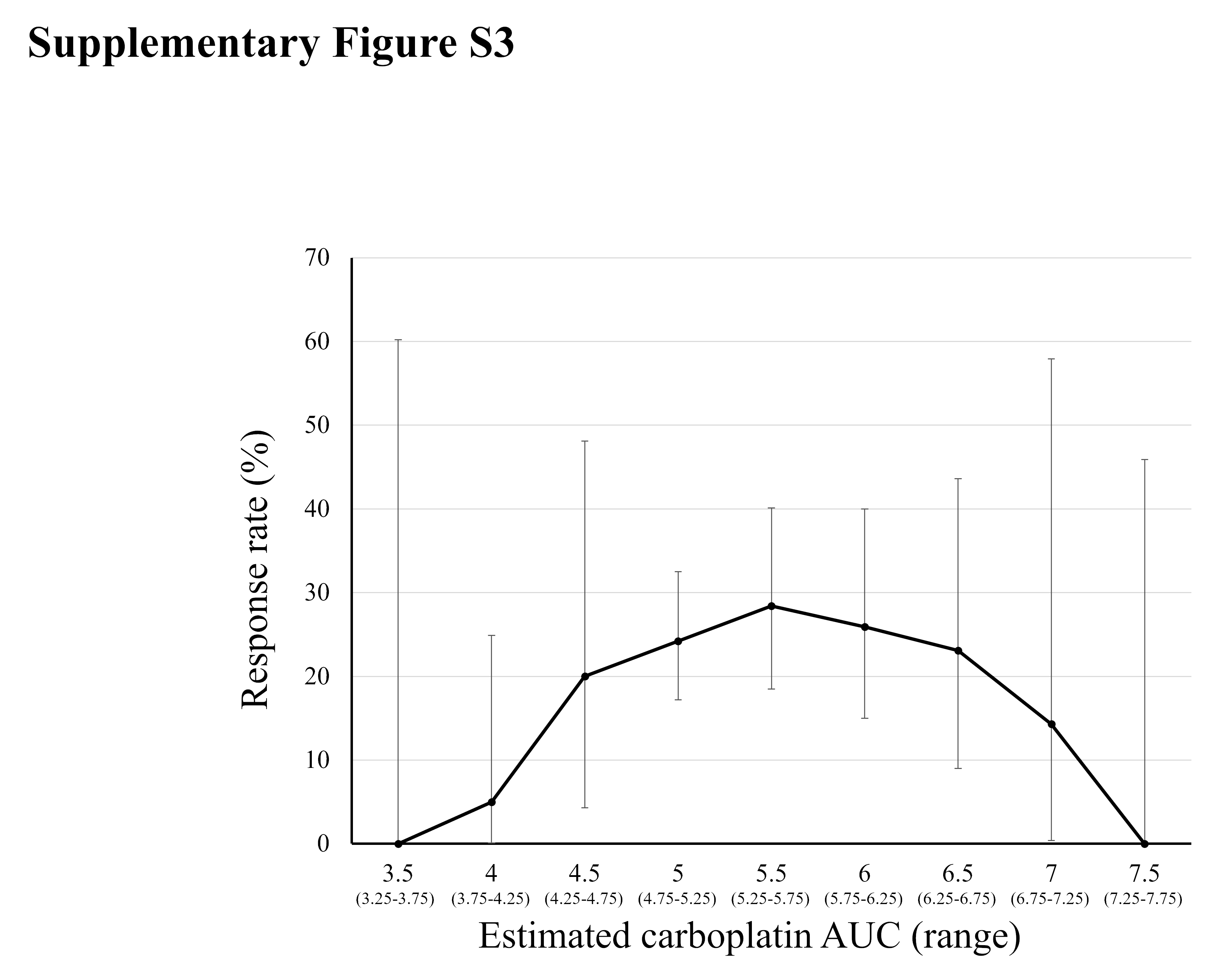

Supplement: Supplementary file 3 — Figure S3. [file CAM4-12-15955-s003.tif]

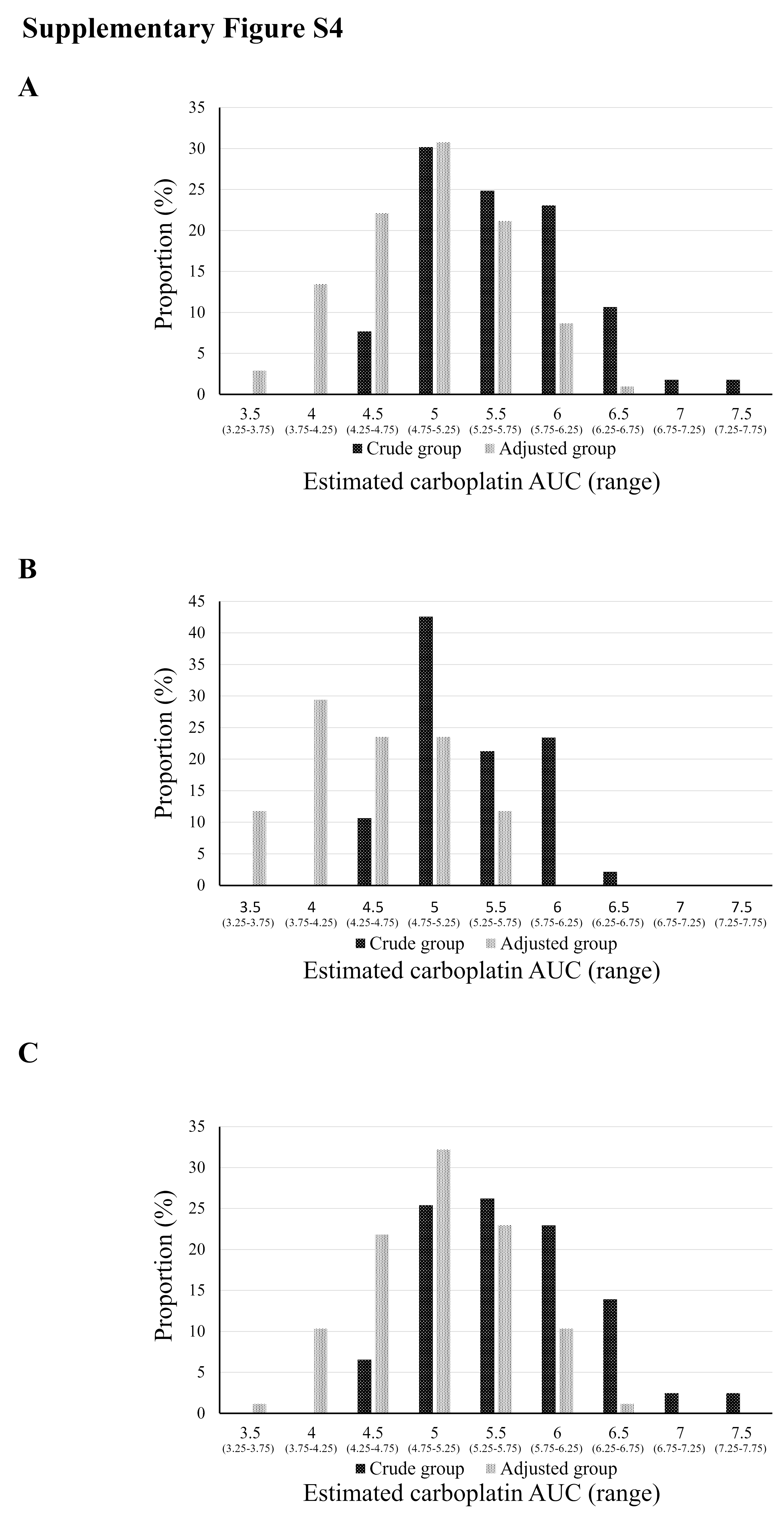

Supplement: Supplementary file 4 — Figure S4. [file CAM4-12-15955-s006.tif]
