# Supplementary material for: Adjustment of creatinine clearance for carboplatin dosing in Calvert's formula and clinical efficacy for lung cancer
Source: Cancer Med. 2023 Jun 23;12(15):15955–69. doi: 10.1002/cam4.6235 (PMC10469651; doi:10.1002/cam4.6235)
Supplement: Supplementary file 5 — Table S1. [file CAM4-12-15955-s004.docx]

**Supplementary Information**

**Adjustment of creatinine clearance for carboplatin dosing in Calvert's formula and clinical efficacy for lung cancer**

Takahiro Hatta^1^, Tetsunari Hase^1*^, Toru Hara^2^, Tomoki Kimura^3^, Eiji Kojima^4^, Takashi Abe^5^, Yoshitsugu Horio^6^, Yasuhiro Goto^7^, Naoya Ozawa^1^, Naoyuki Yogo^1^, Hirofumi Shibata^1^, Tomoya Shimokata^8^, Tetsuya Oguri^9^, Masashi Yamamoto^10^, Kiyoshi Yanagisawa^11^, Masahiko Ando^12^, Yuichi Ando^8^, Masashi Kondo^7^, Makoto Ishii^1^, Yoshinori Hasegawa^1,13^

^1^Department of Respiratory Medicine, Nagoya University Graduate School of Medicine, Nagoya, Japan

^2^Department of Respiratory Medicine, Anjo Kosei Hospital, Anjo, Japan

^3^Department of Respiratory Medicine and Allergy, Tosei General Hospital, Seto, Japan

^4^Department of Respiratory Medicine, Komaki City Hospital, Komaki, Japan

^5^Department of Respiratory Medicine, Ogaki Municipal Hospital, Ogaki, Japan

^6^Department of Thoracic Oncology, Aichi Cancer Center Hospital, Nagoya, Japan

^7^Department of Respiratory Medicine, Fujita Health University School of Medicine, Toyoake, Japan

^8^Department of Clinical Oncology and Chemotherapy, Nagoya University Hospital, Nagoya, Japan

^9^Department of Respiratory Medicine, Allergy and Clinical Immunology, Nagoya City University Graduate School of Medical Sciences, Nagoya, Japan

^10^Department of Respiratory Medicine, Nagoya Ekisaikai Hospital, Nagoya, Japan

^11^Division of Molecular and Cancer Medicine, Faculty of Pharmacy, Meijo University, Nagoya, Japan

^12^Center for Advanced Medicine and Clinical Research, Nagoya University Hospital, Nagoya, Japan

^13^National Hospital Organization, Nagoya Medical Center, Nagoya, Japan

***Correspondence**: Dr. Tetsunari Hase

Department of Respiratory Medicine, Nagoya University Graduate School of Medicine

65 Tsurumai-cho, Showa-ku, Nagoya 466-8550, Japan

ORCID: 0000-0002-9653-8424

Tel: +81-52-744-2167

Fax: +81-52-744-2176

E-mail: [thase@med.nagoya-u.ac.jp](mailto:thase@med.nagoya-u.ac.jp)

**Supplementary Table S1. Odds ratio of hematological toxicity**

|  | Neutrophil count decreased (Grade 3 or 4) | | Anemia (Grade 3 or 4) | | | Platelet count decreased (Grade 3 or 4) | | |
| --- | --- | --- | --- | --- | --- | --- | --- | --- |
| Clinical variable | Odds ratio (95% CI) | *P*-value^a^ | | Odds ratio (95% CI) | *P*-value^a^ | | Odds ratio (95% CI) | *P*-value^a^ |
| eCCr |  |  | |  |  | |  |  |
| Crude group | 1.91 (1.11–3.30) | 0.020 | | 1.56 (0.89–2.76) | 0.124 | | 1.58 (0.92–2.71) | 0.097 |
| Adjusted group | 1 (ref) |  |  | 1 (ref) |  |  | 1 (ref) |  |
| Initial AUC |  |  | |  |  | |  |  |
| 6 | 1.64 (0.98–2.75) | 0.060 | | 1.16 (0.68–1.99) | 0.589 | | 1.34 (0.80–2.25) | 0.260 |
| Less than 6 | 1 (ref) |  |  | 1 (ref) |  |  | 1 (ref) |  |

^a^*P*-values were calculated using logistic regression analysis.

Abbreviations: AUC, area under the blood concentration-time curve; CI, confidence interval; eCCr, estimated creatinine clearance; ref, reference

**Supplementary Table S2. Hematological toxicity in the older and non-older patients**

| Older patients | Crude group (*N* = 47) | |  | Adjusted group (*N* = 17) | |
| --- | --- | --- | --- | --- | --- |
| Adverse event, *N* (%) | Any grade | Grade 3 or 4 |  | Any grade | Grade 3 or 4 |
| Neutrophil count decreased | 40 (85.1) | 19 (40.4) |  | 11 (64.7) | 3 (17.6) |
| Anemia | 47 (100) | 14 (29.8) |  | 16 (94.1) | 2 (11.8) |
| Platelet count decreased | 38 (80.9) | 15 (31.9) |  | 13 (76.5) | 3 (17.6) |
|  |  |  |  |  |  |
| Non-older patients | Crude group (*N* = 122) | |  | Adjusted group (*N* = 87) | |
| Adverse event, *N* (%) | Any grade | Grade 3 or 4 |  | Any grade | Grade 3 or 4 |
| Neutrophil count decreased | 79 (64.8) | 47 (38.5) |  | 58 (66.7) | 25 (28.7) |
| Anemia | 112 (91.8) | 39 (32.0) |  | 82 (94.3) | 22 (25.3) |
| Platelet count decreased | 90 (73.8) | 47 (38.5) |  | 73 (83.9) | 26 (29.9) |
