# Supplementary material for: Adjustment of creatinine clearance for carboplatin dosing in Calvert's formula and clinical efficacy for lung cancer
Source: Cancer Med. 2023 Jun 23;12(15):15955–69. doi: 10.1002/cam4.6235 (PMC10469651; doi:10.1002/cam4.6235)
Supplement: Supplementary file 6 — Legends [file CAM4-12-15955-s005.docx]

**List of supporting information:**

**Supplementary Fig. S1**

A comparison between the bGFR and eCCr in the crude group (A), (B), adjusted group (C), (D), and unclassified patients (E), (F)

Abbreviations: bGFR, back-calculated GFR; eCCr, estimated creatinine clearance; MAE, mean absolute error; RMSE, root mean squared error; SE, standard error

**Supplementary Fig. S2**

Objective response and disease control rate in the adjusted and crude groups and unclassified patients

Abbreviations: DCR, disease control rate; ORR, objective response rate

**Supplementary Fig. S3**

Response rate and estimated carboplatin AUC

Error bar indicates 95% confidence interval.

Abbreviations: AUC, area under the blood concentration-time curve

**Supplementary Fig. S4**

Estimated carboplatin AUC in the older and non-older patients

Proportion of the patients according to indicated estimated carboplatin AUC in all (*N* = 273) (A), older (*N* = 64) (B), and non-older patients (*N* = 209) (C).

Abbreviations: AUC, area under the blood concentration-time curve

**Supplementary Table S1. Odds ratio of hematological toxicity**

**Supplementary Table S2. Hematological toxicity in the older and non-older patients**
